# The effect of 6 Hz transcranial alternating current stimulation over the prefrontal cortex on reward learning in men with methamphetamine use disorder: A pilot randomised double‐blind trial

**DOI:** 10.1002/gps3.70004

**Published:** 2026-03-27

**Authors:** Qianlan Yin, Tianzhen Chen, Yan Long, Daqing Shi, Taosheng Liu, Min Zhao, Jiang Du

**Affiliations:** ^1^ Department of Psychology Naval Medical University Shanghai China; ^2^ Department of Radiology The Second Affiliated Hospital of Naval Medical University Shanghai China; ^3^ Shanghai Jiao Tong University School of Medicine Affiliated Shanghai Mental Health Center Shanghai China; ^4^ Shanghai Drug Rehabilitation Center Shanghai China

**To the editor:**

Methamphetamine use disorder (MUD) is associated with deficits in cognitive control, decision‐making and reward processing, which can contribute to the maintenance of addictive behaviours.^1^ Substantial evidence from neuroimaging, neuropsychological and computational modelling studies also indicates dysregulation of the neural circuits underlying reward processing.^2^, ^3^ Reward learning, crucial in reward processing, may be impaired in MUD, affecting individuals' ability to learn action values and guide goal‐directed behaviours, potentially contributing to substance use disorders.^4^ Recently, there has been growing interest in computational neuroscience approaches to understanding the mechanisms of reward learning deficits in addiction.^5^ Reinforcement learning models have been used to parse the specific components of reward learning that may be impaired in addiction, such as value representation, prediction error signalling and decision‐making.

Neuroimaging research has also identified potential neural mechanisms underlying deficits in reward learning associated with addiction, focusing on the role of the dorsolateral prefrontal cortex (dlPFC) and medial prefrontal cortex (mPFC). The dlPFC is believed to play a role in the hierarchical regulation of subcortical regions associated with reward processing, including areas involved in the evaluation and anticipation of rewards.^6^ Studies have also shown that chronic methamphetamine abuse is associated with structural and functional abnormalities in the dlPFC, which could contribute to the reward processing and decision‐making deficits observed in this population.^7^, ^8^ Anatomically adjacent to the dlPFC, the mPFC is also recognised as a crucial region in reward‐related processes.^9^ It shows graded neural activity across axes, reflecting dimensionality reduction for context processing and decision‐making.^10^, ^11^ Considering this evidence, interventions targeting the PFC regions, including the dlPFC and mPFC, may represent a promising approach to ameliorate reward‐related deficits in MUD.

Although prior neuroimaging studies have explored reward learning deficits in addiction, high‐density electroencephalography (EEG) offers unique insights into the dynamic neural correlations of learning processes. For example, studies have shown that feedback‐related negativity (FRN), an event‐related potential (ERP) associated with reward prediction errors (RPEs), is a reliable neural marker of reinforcement learning.^12^, ^13^ Theta oscillations (4–8 Hz) in the PFC have also been linked to the encoding of reward values and the dynamics of updating expectations during learning.^13^ Current literature shows that dysfunctional theta oscillations may be implicated in the reward processing deficits observed in substance use disorders.^14^ Nevertheless, the prospect of leveraging theta oscillations to develop interventions that ameliorate executive dysfunction in individuals with MUD merits further research and exploration.

Transcranial alternating current stimulation (tACS) is a non‐invasive neuromodulation technique that uses weak alternating current to entrain and modulate neural oscillations and cognitive processes.^15^ A growing body of research has demonstrated that tACS can enhance various cognitive functions, including reward learning, decision‐making and cognitive control.^16^, ^17^ tACS applied to the PFC has shown promise in modulating reward‐related processing and cognitive control in healthy individuals as well as in preliminary studies of addiction.^18^ Importantly, the effects of tACS are target‐ and frequency‐specific, with distinct stimulation frequencies targeting unique cognitive and neurophysiological processes. However, limited research has explored the application of theta‐frequency tACS targeting the PFC to remediate cognitive control deficits in individuals with MUD.

This study used a randomised, double‐blind, sham‐controlled design. The sample included 22 male participants diagnosed with MUD and 21 healthy controls (HC). Participant characteristics and the power analysis are presented in the first section of Supporting Information S1. Participants with MUD received either active or sham 6 Hz tACS during a probabilistic reward learning task. Pre‐intervention assessments were conducted on Day 1, including questionnaires, a behavioural task and electroencephalographic recordings. Participants with MUD were then randomly assigned to either a tACS intervention group or a sham stimulation control group by an independent researcher using a random number table to ensure allocation concealment. The active group received tACS at 6 Hz with an intensity of 1.5 mA peak‐to‐peak for 20 min per session. The electric current gradually ramped up over the first 10 s at the start of stimulation and gradually ramped down over the final 10 s. In the sham group, participants only felt the current during these initial and final 10‐s intervals, as the current remained constant at a minimal level throughout the remaining stimulation period. Details about the intervention protocol are listed in the second section of Supporting Information S1. Interventions were administered twice daily over 2 weeks. Post‐intervention assessment and 1‐month follow‐up evaluated task performance and EEG measurements to assess intervention effects. Ethical approval was obtained from the Ethics Committee of Shanghai Mental Health Center (Ethics Approval No.: 2022‐18C1) in accordance with the principles delineated in the Declaration of Helsinki.

To assess reward learning, participants completed questionnaires and a probabilistic reward task. Primary endpoints included behavioural performance in reward‐related decision‐making. Secondary endpoints included computational modelling parameters and electrophysiological measures to provide mechanistic insights and neural biomarkers. The investigators conducting the statistical tests were unaware of the group assignments until all analyses were completed. Details of these assessments and the statistical analysis are provided in the third section of Supporting Information S1.

Results showed that the MUD group tended to make faster decisions (*z* = −5.032; *p* = 0.008, figure S4A). Computational model‐based analyses (figure S3B) revealed that the MUD group exhibited less model‐based and more model‐free learning than the HC group, as indicated by the lower mixture weight parameter (*z* = −3.181; *p* = 0.002) and a larger inverse temperature parameter (*z* = 7.723, *p* = 0.002). This finding suggests that the MUD group relied more on simple stimulus–response associations and less on flexible, goal‐directed decision‐making.

Figure 1 presents an analysis of ERPs and time‐frequency (TF) responses to reward feedback, comparing HC and MUD groups across positive (RPE+) and negative (RPE−) reward prediction errors. The ERP findings showed that the MUD group had a similar FRN component to the HC group in response to feedback at all central channels (see figure S5 in the five sections of Supporting Information S1). The MUD group exhibited a diminished P300 component at the Cz electrode site in response to negative feedback. Conversely, the TF analysis revealed that the MUD group demonstrated enhanced frontal and central theta oscillatory activity in response to reward feedback, particularly at the Fpz, Cz and CPz electrode sites, compared to the HC group. This indicates that although individuals with MUD can detect prediction errors, the subsequent stages of processing crucial for learning and behavioural adjustment may be compromised.

**FIGURE 1:**
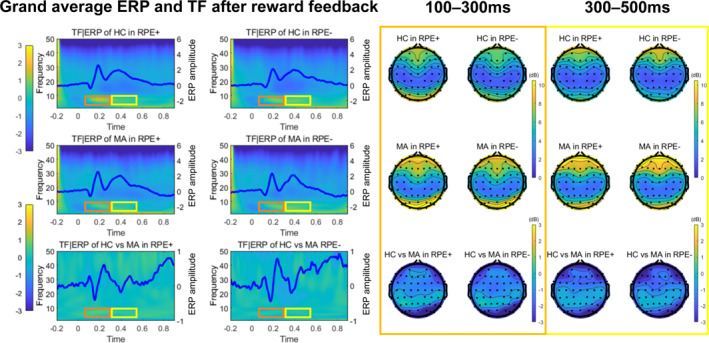
Comparisons of ERP and TF characteristics for RPE between HC and MUD individuals. In the left panel, the top row, ‘HC in RPE+ versus RPE−’ demonstrates that HCs exhibit distinct amplitude and frequency responses to positive and negative feedback. The middle row presents the differences in feedback processing in the MUD group. The bottom row compares ERP and TF responses, highlighting distinct neural mechanisms in response to reward feedback between HCs and the MUD group. The right panel depicts topographic maps illustrating the spatial distribution of ERP amplitudes. The earlier window captures initial processing, whereas the later window reflects integrative feedback processing. The observed variations in ERP amplitudes indicate significant differences in feedback processing between the groups, with colour gradients representing response strength. ERP, event‐related potentials; HC, healthy control; MUD, methamphetamine use disorder; RPE−, negative reward prediction errors; RPE+, positive reward prediction errors; TF, time‐frequency.

The single‐trial analyses further showed that the frontal and central theta power of the HC group was significantly associated with the magnitude of RPEs for both positive and negative feedback (figure S6A, B), as evidenced by the significant non‐zero beta values (continuous *p* < 0.05). In contrast, this relationship was attenuated in the MUD group, with theta power exhibiting a weaker correlation with RPEs. Following positive feedback, the beta activity showed a slight increase in the MUD group compared to the HC group at specific time points, particularly at the Fpz and Fz electrodes. At 350 ms after receiving negative feedback, the MUD group exhibited a marked reduction in beta activity at the Cz electrode site (continuous *p* < 0.05, figure S6B). Conversely, the HC group maintained relatively higher beta levels at Cz and CPz, suggesting that the MUD group may have a diminished neural response to negative prediction errors. However, overall statistical tests did not find significant differences between the groups in how theta oscillations and RPEs related on a single‐trial basis. Despite this overall result, the specific findings regarding reduced beta at Cz after negative feedback show clear differences between the groups. These specific changes, combined with altered beta responses to positive and negative feedback, suggest that in MUD, the link between theta oscillations and RPEs is weakened. This neural profile might explain the consistent maladaptive decision‐making and high relapse rates seen in individuals with MUD.

The active‐treatment group exhibited a significantly greater reduction in Barratt Impulsiveness Scale scores compared to the sham group, as observed after the 2‐week intervention (*t* = 4.751, *p* = 0.041) and at the 1‐month follow‐up (*t* = 6.038, *p* = 0.023), whereas no significant differences were observed in other measures (table S2). The behavioural performance and computational model parameters showed significant interaction effects between time and intervention for both the learning rate (*F* = 4.305, *p* = 0.020) and inverse temperature parameters (*F* = 3.585, *p* = 0.037). The estimated marginal means with corresponding standard errors across time for each group are shown in figure S7. Pairwise comparisons of simple effect estimates revealed that the active‐treatment group significantly increased the learning rate (*t* = 2.355, *p* = 0.028) and inverse temperature parameters (*t* = 2.021, *p* = 0.048) from baseline to the 2‐week assessment, whereas no significant changes were observed in the sham group. However, these effects were not maintained at the 1‐month follow‐up.

Given the observed differences between the HC and MUD groups, we analysed changes in theta‐band power at the Cz and CPz electrodes across the assessment time points, as these differences may be more indicative of MUD characteristics. We found slight variations in theta‐band power within the 300–500 ms timeframe at the Cz electrode (*F* = 3.450, *p* = 0.068, figure S8C), indicating a modest rise in power among the active‐treatment group following the 2‐week period. Nevertheless, these changes over time and between groups did not reach statistical significance (comparable patterns are presented in figures S8 and S9).

Furthermore, the single‐trial analysis results shown in figure 2 illustrate that the active‐treatment group exhibited a significant decrease in the slope of the relationship between theta oscillations and RPEs under negative feedback at Cz and CPz after the intervention, but the changes were not statistically significant under positive feedback. The sham group demonstrated a reduced relationship between theta oscillatory activity and RPEs, as evidenced by the significant non‐zero beta coefficients at the Cz electrode following positive feedback after the 2‐week intervention, whereas the active‐treatment group remained stable in this relationship (figure 2A). The active‐treatment group also showed a significant decrease in the relationship between theta oscillations and RPEs under the negative feedback condition at the Cz and CPz electrodes following the intervention (figure 2B). Specifically, this change was observed in the initial segment (100–200 ms) after negative feedback at CPz for both the 2‐week and 1‐month assessments and in the first half of the time segment (100–300 ms) at Cz during the 2‐week assessment. These results suggest that 6 Hz tACS over the right dlPFC may have modulated reward‐related neural dynamics, particularly regarding negative prediction errors.

**FIGURE 2:**
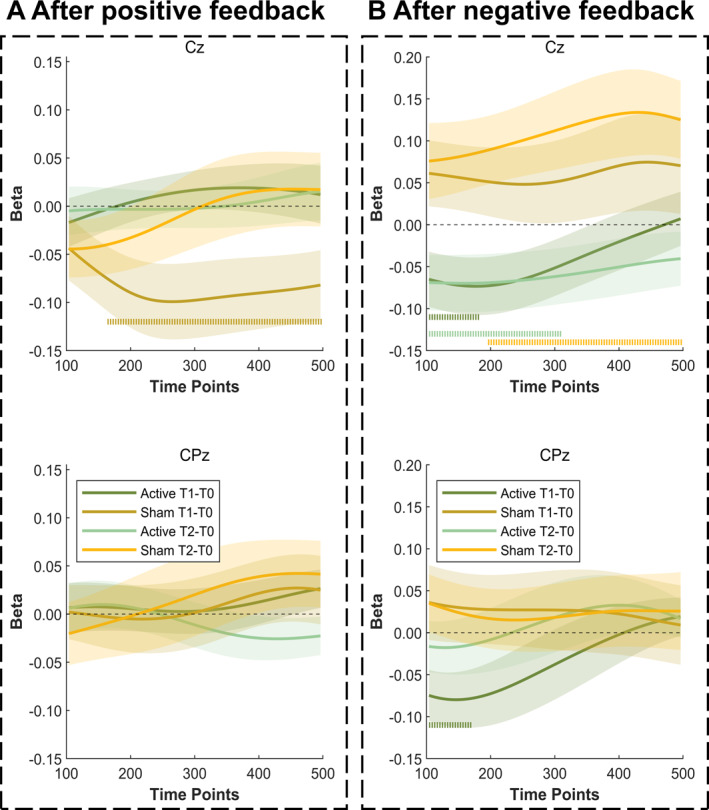
Single‐trial relationship between theta oscillations and reward prediction errors across longitudinal assessments at Cz and Pz electrodes. The lines in each subplot represent the average beta values for the different measure times at various time points, with shaded areas indicating the confidence intervals of the data. The statistical significance of the non‐zero beta coefficients for each group is denoted by horizontal dotted lines. Beta, beta coefficient; T1–T0, 2‐week time point relative to baseline; T2–T0, 1‐month time point relative to baseline.

The present study investigated the effects of 6 Hz tACS over the mPFC and left dlPFC on reward‐related behavioural and neural dynamics. Individuals with MUD showed reduced sensitivity to positive and negative feedback, suggesting a general deficit in flexibly adjusting behaviour based on environmental cues. They employ a more simplistic ‘win‐stay, lose‐shift’ decision‐making strategy and are more likely to switch to an alternative option when their strategy fails. This overreliance on rigid processes and diminished reward sensitivity may explain the persistent use of methamphetamine despite adverse consequences, a hallmark of addiction.

Our results also indicate that the active‐treatment group exhibited significantly improved impulsivity, suggesting enhanced control and flexibility. Importantly, these effects persisted at the 1‐month follow‐up, suggesting that 6 Hz tACS over the mPFC and left dlPFC may stabilise and potentially enhance domain‐general cognitive control mechanisms, which can improve decision‐making performance and reduce compulsive behaviours in individuals with MUD. Besides, the intervention enhanced RPE sensitivity in individuals with MUD, as evidenced by increased learning rate and inverse temperature parameters after 2 weeks of stimulation. However, no significant improvements were observed in model‐based learning, suggesting that 6 Hz tACS primarily modulated the model‐free learning component, specifically enhancing the ability to adjust behaviour based on negative RPEs for individuals with MUD. Consistent with the previous hypothesis, our single‐trial analysis demonstrated that 6 Hz tACS over the mPFC and left PFC elicited changes in the relationship between theta oscillations and RPEs, particularly for negative feedback, at central parietal sites. Therefore, these findings suggest that 6 Hz tACS over the left dlPFC may modulate reward‐related neural dynamics, particularly in negative feedback processing.

Some limitations of the present study should be considered. First, recruitment challenges, including difficulties in sustained participant engagement and managing comorbidities, led to a smaller sample size (44 participants, 22 with MUD) than the preregistered target of 210 participants. This limits the statistical power and generalisability of our findings, though post hoc power analyses were conducted to clarify detectable effect sizes. Therefore, these findings should be considered preliminary and interpreted with caution until replicated in larger, adequately powered cohorts. Second, future research should expand participant demographics to include female participants, a wider range of ages, more diverse socioeconomic backgrounds and different durations of MUD and abstinence periods to enhance generalisability.

Nonetheless, our findings demonstrate that 6 Hz tACS applied over the PFC can selectively enhance RPE‐driven learning and reduce impulsivity in individuals with MUD. These results suggest that directly targeting the oscillatory dynamics in the PFC may be a promising approach to ameliorate the reward‐related behavioural and neural processing deficits observed in MUD.

## AUTHOR CONTRIBUTIONS

Qianlan Yin, Tianzhen Chen, Yan Long, Daqing Shi, Min Zhao and Jiang Du contributed to the methodology and investigation of the study, including study design, participant recruitment, data collection and implementation of the tACS and behavioural tasks. Qianlan Yin took primary responsibility for data curation, formal analysis and drafting of the original manuscript. Tianzhen Chen, Taosheng Liu, Min Zhao and Jiang Du contributed to the review and critical revision of the manuscript for important intellectual content and assisted in refining the interpretation of findings. Min Zhao and Jiang Du were responsible for funding acquisition, resource provision, project administration, supervision and overall conceptualisation of the research. Jiang Du acted as guarantor. All authors approved the final version of the manuscript and agreed to be accountable for all aspects of the work, ensuring the accuracy and integrity of the study.

## FUNDING

This work was supported by the National Natural Science Foundation of China (Grant Nos. 8257055532 [Jiang Du], 82571704 [Tianzhen Chen], 82201650 [Tianzhen Chen], 82130041 [Min Zhao]); the Shanghai Jiao Tong University Medical Engineering Cross Research Fund (Grant No. YG2023ZD25 [Jiang Du]); the Collaborative Innovation Cluster Project of the Shanghai Municipal Health Commission (Grant No. 2024CXJQ03 [Jiang Du]); the Shanghai ‘Rising Stars of Medical Talents’ Youth Development Program (Grant No. SHWSRS(2025)_071 [Tianzhen Chen]); and the Clinical Research Projects of the Shanghai Municipal Health Commission (Grant No. 20244Y0201 [Tianzhen Chen]).

## Supporting information

Supporting Information S1
