# Supplementary material for: The effect of 6 Hz transcranial alternating current stimulation over the prefrontal cortex on reward learning in men with methamphetamine use disorder: A pilot randomised double‐blind trial
Source: Gen Psychiatr. 2026 Mar 27;39(1):e70004. doi: 10.1002/gps3.70004 (PMC13064579; doi:10.1002/gps3.70004)
Supplement: Supplementary file 1 — Supporting Information S1 [file GPS3-39-e70004-s001.docx]

1. **Enrollment and protocol**

**1.1 Participants**

A total of 110 individuals were screened from the rehabilitation organisation; 88 did not meet inclusion criteria or declined participation, leaving 22 participants who were enrolled in the study (see Figure 1). The final sample included 22 male participants diagnosed with MUD. Inclusion criteria for the MUD group were: 1) meeting diagnostic criteria for MUD according to the Diagnostic and Statistical Manual of Mental Disorders, 5th edition (DSM-5); 2) having used methamphetamine at least twice per week on average in the past 6 months; 3) no history of other substance use disorders (except nicotine dependence) according to DSM-5; 4) no current or past neurological disorders (eg, epilepsy, stroke) or major psychiatric disorders (eg, schizophrenia, major depressive disorder) other than MUD. Exclusion criteria included a history of head injury with loss of consciousness for more than 30 minutes, a history of seizures, any medical condition potentially impacting brain function (eg, severe heart, liver or kidney disease) and contraindications to tACS (eg, presence of a pacemaker).

The healthy control (HC) group, consisting of 22 males with no psychiatric or neurological disorders, were recruited separately from community populations within the same city as the collaborating rehabilitation organisation to ensure comparable socioeconomic and cultural backgrounds. The age and gender of the HC group were matched to the MUD group, yet one participant dropped out due to EEG recording issues, resulting in a final analysed sample of 43 participants (21 MUD and 22 HC). Detailed demographic characteristics of both groups are provided in Table S1.

Power analysis based on the enrolled sample (n = 43) indicated that, assuming an expected mean difference of 0.5 and a standard deviation of 0.28, there is an almost certain probability (approximately 99.99%) of identifying a statistically significant difference between the two independent groups at a significant level of 5%. Using a repeated-measures ANOVA framework (2 groups × 3 time points, 11 participants in each group) with a correlation among repeated measures of 0.5 and the observed effect size of f = 0.38, the post hoc power analysis indicates a high statistical power of 97.2% for detecting a group × time interaction.


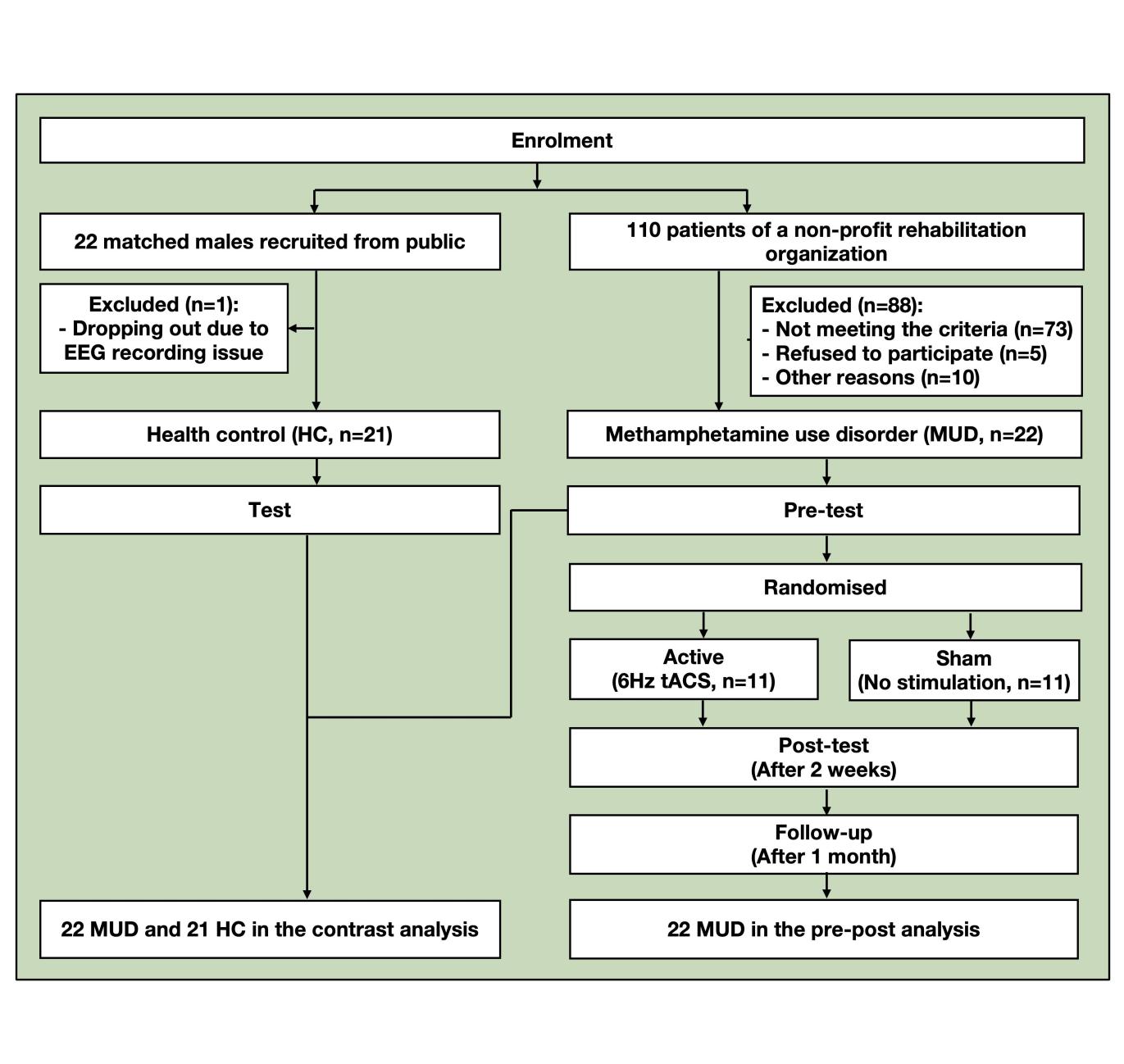


Figure S1 Schema of the data collection and treatment protocol

**1.2 Study protocol**

The study used a randomised, double-blind, sham-controlled design. Individuals with MUD received either active or sham 6 Hz tACS during a probabilistic reward learning task. Pre-intervention assessments were conducted on Day 1, including questionnaires, a behavioural task and electroencephalographic recordings. Participants with MUD were then randomly assigned to either a 6 Hz tACS intervention group or a sham stimulation control group by an independent researcher using a random number table to ensure allocation concealment. The intervention protocol was designed to target the mPFC and left dlPFC using a Focus V3 tES device. The active group received tACS at 6 Hz with an intensity of 1.5 mA peak-to-peak for 20 minutes per session, twice daily. The electric current gradually ramped up over the first 10 seconds at the start of stimulation and gradually ramped down over the final 10 seconds. In the sham group, participants only felt the current during these initial and final 10-second intervals, as the current remained constant at a minimal level throughout the remaining stimulation period. To ensure accurate electrode placement, the left dlPFC was targeted using the 10–20 EEG system. The active electrode was placed over the F3 and Fpz in an alternating polarity configuration. Circular sponges with a 2.2 cm diameter, saturated with physiological saline for conduction, were used, enclosed within a 3.5 cm x 3.5 cm rubber casing. An a-priori simulation of the setup was conducted on the “ernie” dataset available at the SimNIBS site (see Figure S2). The modelling results suggest that the montage could achieve sufficient electric field intensities of around 0.20 V/m in both the mPFC and left dlPFC target regions. Both the real tACS and sham groups reported similar sensations during the stimulation, with no reports of tingling sensations. Participants were seated comfortably in an electrostatically shielded room throughout the tACS stimulation sessions.

**
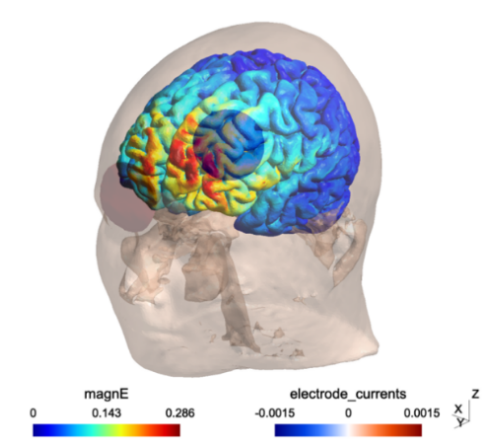
**

Figure S2 A priori simulation of the electric field distribution.

Note. The simulation was conducted on the “ernie” template brain provided by SimNIBS.

Interventions were administered twice daily over two weeks. Post-intervention assessment and one-month follow-up evaluated task performance and EEG measurements to assess intervention effects. Ethical approval was obtained from the Ethics Committee of *****(Ethics Approval No.: 2022-18C1) with the principles delineated in the Declaration of Helsinki. The now-closed trial was prospectively registered at ClinicalTrials.gov (NCT05312359). During the pilot phase, recruiting patients with MUD enabled a more homogeneous sample and was approved by the Ethics Committee. The current dataset, though smaller, offers preliminary insights and informs future research.

1. **Assessments and analyses**
   1. **Questionnaires**

Prior to the experiment, participants were required to complete five validated questionnaires to assess their recent psychological state. The Chinese translation for the Self-Rating Depression Scale and the Self-Rating Anxiety Scale each consist of 20 items rated on a 4-point scale to evaluate the frequency of symptoms over a specified timeframe^45 46^. The Pittsburgh Sleep Quality Index is a 19-item scale that assesses overall sleep quality through seven components^47^. The Perceived Stress Scale is a 10-item measure that gauges participants' perceptions of their lives as unpredictable, uncontrollable and overwhelming^48^. The Barratt Impulsiveness Scale (BIS) is a 30-item instrument that examines different dimensions of impulsivity, encompassing attentional, motor and non-planning aspects^49^. The Addiction-related Clinical Symptom Scale (ACSS) was used to measure various physical and psychological symptoms experienced by individuals with MUD group^50^.

- 1. **The probabilistic reward learning task**

This task consisted of a two-step decision task, adjusted in earlier work by Sambrook and colleagues illustrated in Figure S1 in the supplementary file^51^. Participants began by selecting one of two fractal images at Stage 1 (State1), each associated with a higher probability (70%) of leading to Stage 2 (State2) and a lower probability (30%) of reaching the other. At State2, participants received either a reward or no reward, with reward probabilities for each fractal ranging between 0.25 and 0.75 and fluctuating independently. Participants inferred these probabilities based on feedback and developed valuations for both State1 and State2 choices. A computational model with model-based and model-free learning components was used to assess decision-making, highlighting the systems’ distinct responses to reward feedback and transition probabilities. For a more comprehensive introduction, please refer to the supplementary files. Task performance was preliminarily examined by analysing participants' choice accuracy or task earnings and reaction times.
 As illustrated in Figure S2A, participants engaged in a probabilistic learning structure. They began by selecting one of two fractal images presented at the initial stage (State1). This choice led them to one of two possible intermediary stages (State2), where a single fractal was displayed. Crucially, each fractal at State1 was associated with a higher probability (70%) of one specific State2 and a lower probability (30%) of leading to the other State2. These probabilities were explicitly communicated to the participants. Subsequently, at State2, participants received an outcome, either a reward or no reward. The probabilities of receiving a reward from each State2 fractal were initially set between 0.25 and 0.75 and were allowed to change gradually and independently within those bounds throughout the task. Participants inferred the hidden probabilities associated with each State2 based on the presence or absence of reward feedback. It's important to note that the rewards depended solely on the specific State2 reached, not the initial State1 choice. As the task progressed, participants were expected to develop distinct valuations for the two-state 2 fractals based on their experience with reward outcomes. Furthermore, since the State1 choice influenced the likelihood of reaching a particular State2, participants would also form distinct valuations for the two-state 1 choices. These valuations, for both State1 choices and State2 outcomes, were computationally modeled on a trial-by-trial basis using a model incorporating both model-based and model-free learning components that generate different value estimates. By comparing how well each system's estimation aligned with the observed behaviour, we could assess the extent to which each learning system was driving decision-making. To illustrate why the model-free and model-based valuations diverge in this task, consider a scenario where a participant reaches the State2 fractal via the less likely transition and receives a reward. The model-based and model-free systems would increase the value assigned to that specific State2 fractal. However, when considering the initial State1 choice, the model-based system, due to its knowledge of the task structure, would increase the value of the unchosen option. This is because the model-based system recognises that the unchosen option has a higher probability of leading to the recently rewarded State2. In contrast, lacking this structural understanding, the model-free system would simply increase the value of the chosen State1 fractal because it resulted in a reward on that trial. While this example illustrates a specific scenario, the general principle is that the model-free system's disregard for transitional probabilities, as opposed to the model-based system's incorporation of this information, leads to distinct valuations throughout the task.


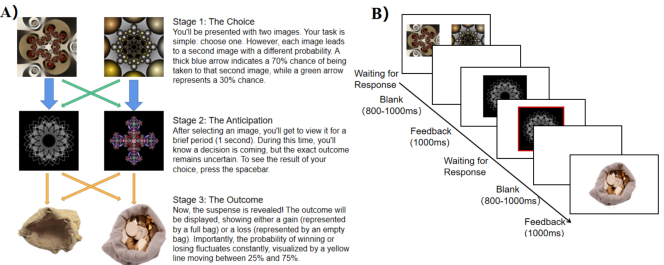


Figure S2 The paradigm of the two-step decision task. A) The task structure presented to the participants is illustrated; B) the electroencephalography data collection design timeline is outlined. The fractal images used in this study were obtained from Pixabay and Pexels, which provide images licensed for free use.

**2.3 Computational models**

A hierarchical Bayesian reinforcement learning model was fitted to the behavioural data to examine the computational mechanisms underlying reward learning. The model included four key parameters: 1. learning rate (α), the degree to which participants updated their value estimates on each trial based on the RPE; 2. inverse temperature (β), the degree to which participants' choices were guided by their value estimates (higher values indicate more deterministic, greedy choices); 3. the mixture weight captured the relative contributions of model-free and model-based learning (ω) and 4. the eligibility parameter (λ), modulating an additional stage-skipping update of the State1 action by the State2 prediction error. The model-free learning component was implemented as a standard Q-learning algorithm, which updates state-action values based on the RPE. The model-based component was implemented using a transition matrix that encoded the probability of transitioning from each State1 to the possible State2 outcomes. Computational modelling allowed us to quantify the degree to which participants relied on model-free versus model-based learning strategies, as reflected by the mixture of weight parameters.

The computational model and the related parameter fitting procedures were executed in R (R Core Team, 2017) utilising the slpMBMF function from the catlearn package (Catlearn Core Team, 2017). This model is built upon the framework used by Gillan et al. (2015) and features distinct components for model-free and model-based learning, which operate simultaneously to generate their individual value estimates for the fractals. In the task, to derive a single overall value for each fractal that will be used for action selection, the estimates from both the model-based and model-free components are averaged, with the weighting reflecting the participant's inclination towards either model-free or model-based learning. This inclination, along with additional parameters, was determined based on the participant's behaviour.

**Model-free component:** The model-free aspect utilised the SARSA(λ) temporal difference learning algorithm (Sutton and Barto, 1998). This algorithm governs learning at both state 1 and state 2 of the task, which can manifest in three forms: the initial state, referred to as s_A_, which is always encountered, and two alternative states, labeled s_B_ and s_C_, with only one being experienced in any given trial(t). At these states, an action, denoted as a, is executed, and a reward, r, is received at the end of the trial. The states and actions for state 1 and state 2 are represented as s_1_, a_1_ and s_2_, a_2_, with the rewards following state 1 and state 2 indicated as r_1_ and r_2_ (where r_1_ is always zero in this task). The model-free value Q_MF for a specific state-action pair is updated using the learning rate α and the model-free prediction error δ_MF_, as follows:

Q_MF_(s_i,t_, a_i,t_) = Q_MF_(s_i,t_, a_i,t_) + α * δ_MF,i,t_

where

δ_MF,i,t_ = r_i,t_ + Q_MF_(s_i+1,t,_ a_i+1,t_) - Q_MF_(s_i,t_, a_i,t_)

This represents the general form of the algorithm. However, in this task, actions are not taken at states s_B_ and s_C_ (thus, the state-action values there are more accurately described as state values). As previously mentioned, rewards are not obtained after s_1_, so δ_MF,1,t_ is solely based on the Q_MF_ value of the state 2 that was transitioned to. Conversely, since no state is reached after state 2, δ_MF,2_ is determined only by the reward r_2_. At the end of the trial, the eligibility parameter λ is applied to adjust an additional stage-skipping update of the state 1 action based on the state 2 prediction error:

Q_MF_(s_1,t_, a_1,t_) = Q_MF_(s_1,t_, a_1,t_) + α * λ * δ_MF,2,t_

**Model-based component**: The model-based learning component relies on established knowledge about how the action taken at state 1 probabilistically influences the state 2 that is reached. It integrates this information with the value of state 2 to calculate action values for state 1. The equation is represented as:

Q_MB_(s_A_, a_j_) = P(s_B_ | s_A_, a_j_) * Q_MF_(s_B_) + P(s_C_ | s_A_, a_j_) * Q_MF_(s_C_)

At state 1, participants can choose between two actions, represented as pressing the left and right keys, denoted as a_A_ and a_B_. The transition probabilities, which participants were aware of, are as follows: P(s_B_ | s_A_, a_A_) = 0.7; P(s_C_ | s_A_, a_B_) = 0.7; P(s_B_ | s_A_, a_B_) = 0.3; and P(s_C_ | s_A_, a_A_) = 0.3.

Hybrid Action Value: For the purpose of action selection, the Q values from both the model-free and model-based components at state 1 are combined into a hybrid value, Q_H_. This hybrid value is a weighted average of the individual values, Q_MF_ and Q_MB_, with the weight determined by **ω**, which reflects the extent to which the participant's behaviour is model-free or model-based. The equation is expressed as:

Q_H_( s_A_, a_j_) = **ω** * Q_MB_( s_A_, a_j_) + (1 - **ω**) * Q_MF_( s_A_, a_j_)

This hybrid value is solely for action selection and does not influence the learning process. In terms of learning, the model-based and model-free components maintain their distinct Q values, which are updated over trials as previously described.

**Parameter fitting**: The parameters **ω, α,** and **λ** were estimated for each participant using maximum likelihood based on their observed choices. This process required the application of a choice rule. A standard softmax rule was employed, incorporating the inverse temperature parameter **β** (which was also fitted) to calculate the probability, P, of each choice at state 1:

P(a_i,t_ = a | s_i,t_) = exp[β * Q_H_(s_i,t_, a)] / Σa' exp[β * Q_H_(s_i,t_, a)]

Each participant's data was fitted individually using the L-BFGS-B method(Byrd et al., 1995) utilised the optim function in R. To enhance the stability of the neural regressors, a second fitting stage was performed, as outlined by Daw et al. (2011). In this stage, all participants were re-fitted with all parameters, except for **ω**, which was fixed at the median value obtained from the first fitting stage.

References:

R Core Team, 2017. R: A Language and Environment for Statistical Computing. https://

[www.r-project.org/.](http://www.r-project.org/.)

Catlearn Core Team, 2017. catlearn: Formal Modeling for Psychology. R package version 0.5. <https://CRAN.R-project.org/package¼catlearn.>

Gillan, C.M., Otto, A.R., Phelps, E.A., Daw, N.D., 2015. Model-based learning protects against forming habits. Cognit. Affect. Behav. Neurosci. 15 (3), 523–536. https://doi.org/10.3758/s13415-015-0347-6.

Sutton, R.S., Barto, A.G., 1998. Introduction to Reinforcement Learning. MIT Press

Daw, N.D., Gershman, S.J., Seymour, B., Dayan, P., Dolan, R.J., 2011. Model-based influences on humans' choices and striatal prediction errors. Neuron 69 (6), 1204–1215. <https://doi.org/10.1016/j.neuron.2011.02.027.>

Byrd, Richard H., et al., 1995. A limited memory algorithm for bound constrained optimisation. SIAM Journal on Scientific Computing 16.5, 1190–1208.

**4. Demographic characteristics, addiction history and psychological assessment scores**

Table S1. Demographic characteristics, addiction history and psychological assessment scores of the HC and MUD groups

| Characteristic | HC  (n=21) | MUD  (n=22) | *t* | *P* |
| --- | --- | --- | --- | --- |
| Age | 41.67(10.30) | 39.00(9.92) | 0.748 | 0.392 |
| Education | 10.10(2.17) | 10.64(4.76) | 0.227 | 0.636 |
| Years of addiction | - | 5.59(4.35) | - | - |
| Months of abstention | - | 30.14(24.28) | - | - |
| ACSS | - | 5.55(5.32) | - | - |
| SDS | 36.57(6.03) | 40.31(10.05) | 2.100 | 0.155 |
| SAS | 38.24(6.80) | 42.15(9.92) | 2.188 | 0.147 |
| PSQI | 6.76(2.95) | 8.10(4.01) | 1.491 | 0.229 |
| PSS | 14.19(5.53) | 13.73(7.45) | 0.050 | 0.824 |
| BIS | 33.58(15.03) | 39.86(20.02) | 1.298 | 0.261 |

Note: ACSS=Addiction-related Clinical Symptom Scale; BIS=Barratt Impulsiveness Scale; HC=healthy control; MUD=methamphetamine use disorder; PSQI=Pittsburgh Sleep Quality Index; PSS=Perceived Stress Scale; SAS=Self-Rating Anxiety Scale; SDS=Self-Rating Depression Scale.

**5. Behavioural results plot**

Figure S4A illustrates how participants' inferred probabilities of receiving rewards evolved over 200 trials in a task where rewards were associated solely with the specific State2 outcomes, independent of their initial State1 choices. Notably, the pink diamonds and purple triangles represent the predefined probabilities of outcomes following State2, highlighting the specific reward structures participants navigated throughout the trials.


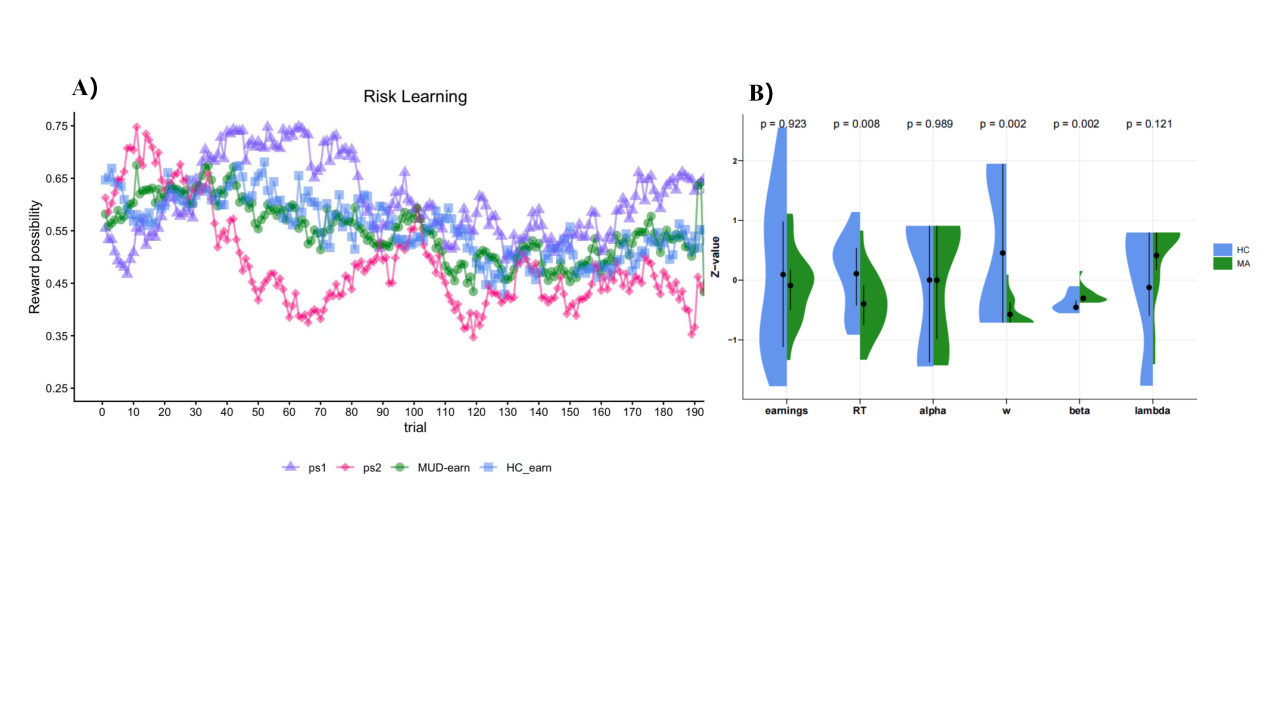


Figure S4 Behavioural performances and computational model parameters. A) The Y-axis represents reward possibility (ranging from 0.25 to 0.75), while the X-axis indicates trial number. Data points in different colors (green circles, pink diamonds, blue squares, and purple triangles) reflect distinct conditions. Pink diamonds and purple triangles represent the predefined possibility of outcome after State2. Green circles and blue squares represent the group-average reward possibility for MUD, respectively. B) Each behavioural indexes and parameters are represented on the x-axis, while the Z-values are plotted on the y-axis. The areas shaded in blue and green correspond to HC and MUD groups, respectively, illustrating the distribution of Z-values.

1. **ERP components and theta oscillatory dynamics in MUD compared to HC**

**6.1 Plotting**


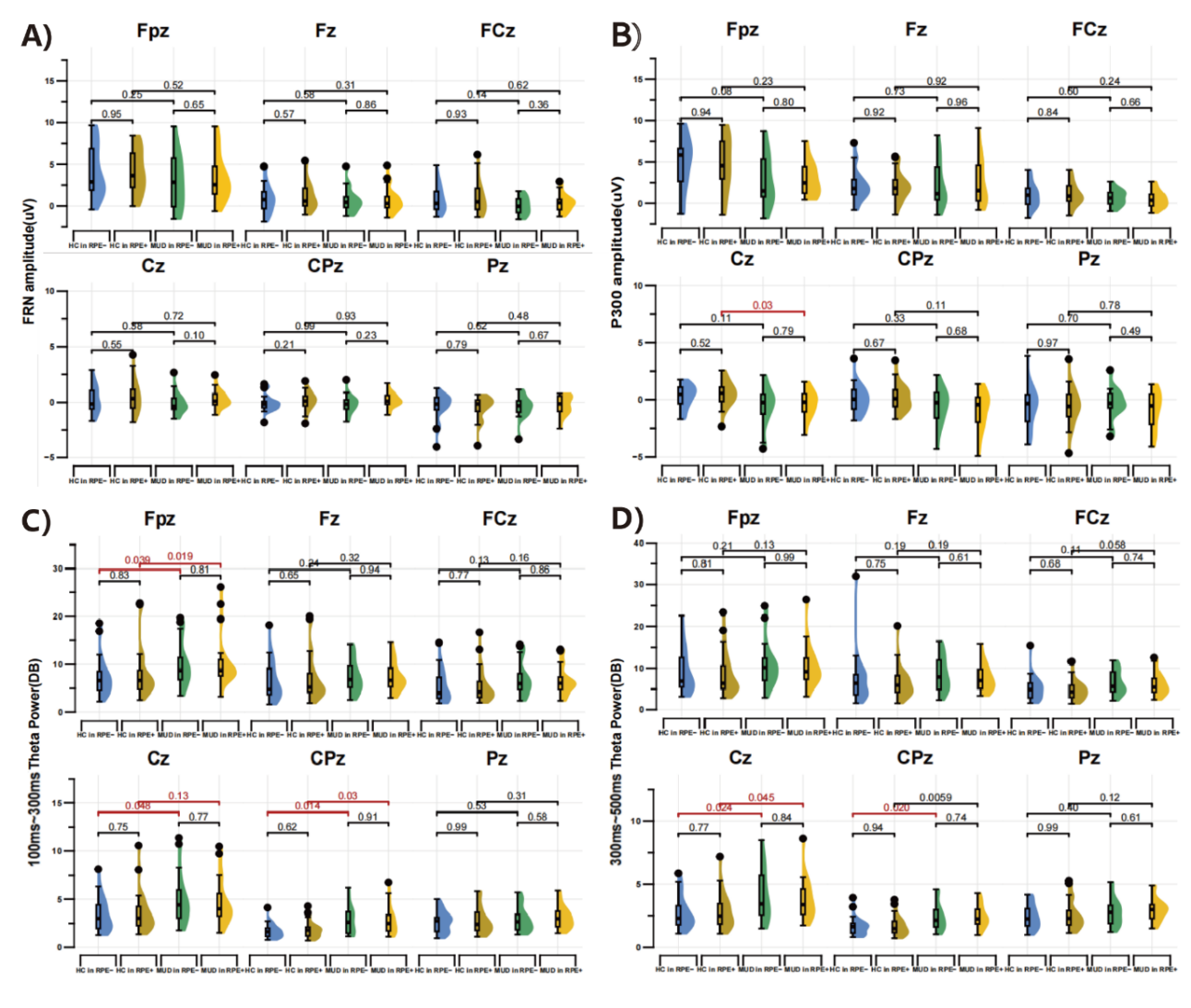


Figure S5 Comparative Analysis of FRN and P300 Amplitude and Time-Frequency Power across Electrode Sites. A) and B) present violin plots displaying the Feedback Related Negativity (FRN) amplitudes and P300 components measured at various EEG channels (FCz, Fz, Fpz, Cz, CPz, and Pz). C) and D) illustrates the theta band power changes at the electrode sites Fpz, Cz, and FCz over two time intervals. The violin plots depict the distribution of power values, with significant differences indicated by the p-values above the comparisons. P-values less than 0.05 are highlighted in red, signifying statistically significant differences.

**6.2 ANOVA results for FRN, P300, and theta power in HC and MUD groups**

*ANOVA results using value as the FRN*

Predictor SS df MS F p partial_eta2 CI_90_partial_eta2

(Intercept) 1.08 1 1.08 0.08 .777

condition 0.68 1 0.68 0.05 .822 .00 [.00, .01]

MUD 4.23 1 4.23 0.31 .575 .00 [.00, .01]

channel 773.20 5 154.64 11.50 .000 .11 [.06, .15]

condition x MUD 0.00 1 0.00 0.00 .996 .00 [.00, 1.00]

condition x channel 3.12 5 0.62 0.05 .999 .00 [.00, 1.00]

MUD x channel 119.27 5 23.85 1.77 .117 .02 [.00, .03]

condition x MUD x channel 0.17 5 0.03 0.00 1.00 .00 [.00, 1.00]

Error 6131.97 456 13.45

*ANOVA results using value as the P300*

Predictor SS df MS F p partial_eta2 CI_90_partial_eta2

(Intercept) 0.07 1 0.07 0.00 .960

condition 0.27 1 0.27 0.01 .921 .00 [.00, .00]

MUD 9.57 1 9.57 0.35 .557 .00 [.00, .01]

channel 1256.66 5 251.33 9.10 .000 .09 [.05, .13]

condition x MUD 0.05 1 0.05 0.00 .966 .00 [.00, 1.00]

condition x channel 6.54 5 1.31 0.05 .999 .00 [.00, 1.00]

MUD x channel 303.69 5 60.74 2.20 .053 .02 [.00, .04]

condition x MUD x channel 1.88 5 0.38 0.01 1.00 .00 [.00, 1.00]

Error 12597.83 456 27.63

*ANOVA results using value as the 100ms-300ms Theta power*

Predictor SS df MS F p partial_eta2 CI_90_partial_eta2

(Intercept) 1225.66 1 1225.66 116.29 .000

condition 5.33 1 5.33 0.51 .477 .00 [.00, .01]

MUD 69.38 1 69.38 6.58 .011 .01 [.00, .04]

channel 576.46 5 115.29 10.94 .000 .11 [.06, .14]

condition x MUD 0.00 1 0.00 0.00 .994 .00 [.00, 1.00]

condition x channel 3.23 5 0.65 0.06 .998 .00 [.00, 1.00]

MUD x channel 31.55 5 6.31 0.60 .701 .01 [.00, .01]

condition x MUD x channel 2.88 5 0.58 0.05 .998 .00 [.00, 1.00]

Error 4806.29 456 10.54

*ANOVA results using value as the 300ms-500ms Theta power*

Predictor SS df MS F p partial_eta2 CI_90_partial_eta2

(Intercept) 1840.38 1 1840.38 121.58 .000

condition 1.61 1 1.61 0.11 .745 .00 [.00, .01]

MUD 110.63 1 110.63 7.31 .007 .02 [.00, .04]

channel 1007.15 5 201.43 13.31 .000 .13 [.08, .17]

condition x MUD 1.56 1 1.56 0.10 .748 .00 [.00, .01]

condition x channel 9.23 5 1.85 0.12 .987 .00 [.00, 1.00]

MUD x channel 53.71 5 10.74 0.71 .616 .01 [.00, .01]

condition x MUD x channel 2.59 5 0.52 0.03 .999 .00 [.00, 1.00]

Error 6902.83 456 15.14

**7. Single-trial relationship between theta oscillations and RPEs in MUD and HC groups**


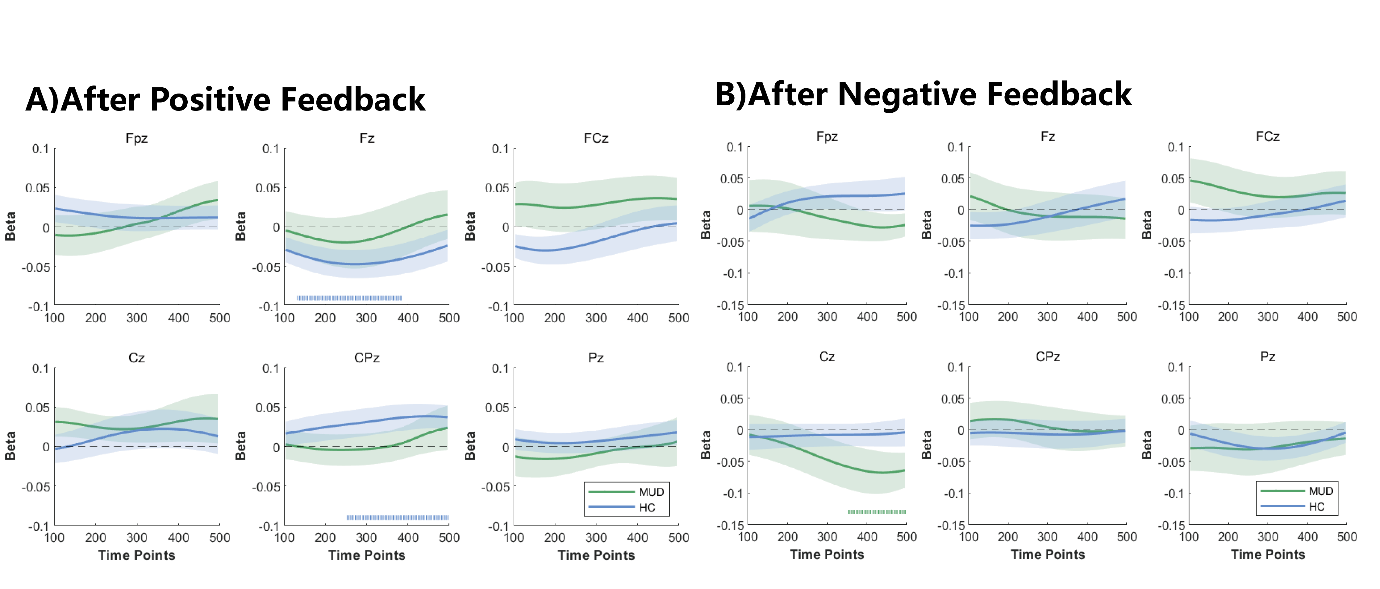


Figure S6 Examination of Single-Trial Theta Oscillatory Dynamics to Reward Prediction Errors Across Central Electrodes. A) illustrates the results following positive prediction, depicting how TF powers vary across electrode sites over time. B) showcases the results after negative predictions. The shaded areas indicate the standard error of the mean for both groups. The statistical significance of the non-zero beta coefficients for each group is denoted by the dotted lines, colored according to the corresponding group (i.e., green for the MUD group, blue for the HC group).

**8. Self-reported changes in psychological and behavioural functioning**

Table S2 Demographic and clinical characteristics of the active and sham stimulation subgroups.

| Characteristic | Overall | Active | Sham | t | P |
| --- | --- | --- | --- | --- | --- |
| N | 22 | 11 | 11 |  |  |
| Age | 39.00(9.92) | 40.18(9.10) | 37.82(10.99) | 0.302 | 0.589 |
| Education | 10.64(4.76) | 9.55(4.66) | 11.73(4.82) | 1.166 | 0.293 |
| Years of addiction | 5.59(4.35) | 6.45(4.80 | 4.73(3.88) | 0.862 | 0.364 |
| Months of abstention | 30.14(24.28) | 35.09(26.06) | 25.18(22.48) | 0.912 | 0.351 |
| ACSS T1−T0 | 0.14(5.23) | 0.55(6.70) | −0.27(3.50) | 0.129 | 0.723 |
| SDS T1−T0 | 0.59(8.69 | 0.09(10.11) | 1.09(7.46) | 0.070 | 0.795 |
| PSS T1−T0 | 2.02(8.23) | 3.84(8.39) | 0.19(8.04) | 1.084 | 0.310 |
| BIS T1−T0 | 0.48(3.68) | −1.09(2.51) | 2.06(4.09) | 4.751 | 0.041 |
| ACSS T2−T0 | −2.00(3.87) | −3.00(4.40) | −1.00(3.13) | 1.507 | 0.234 |
| SDS T2−T0 | −2.23(10.59 | −5.82(8.49) | 1.36(11.61) | 2.741 | 0.113 |
| PSS T2−T0 | 1.65(9.15) | 1.11(8.82) | 2.19(9.87) | 0.073 | 0.789 |
| BIS T2−T0 | 0.98(3.64) | −0.73(3.50) | 2.70(3.02) | 6.038 | 0.023 |

Note: ACSS=Addiction-related Clinical Symptom Scale; BIS=Barratt Impulsiveness Scale; HC=healthy control; MUD=methamphetamine use disorder; PSQI=Pittsburgh Sleep Quality Index; PSS=Perceived Stress Scale; SAS=Self-Rating Anxiety Scale; SDS=Self-Rating Depression Scale.. Furthermore, the terms T1−T0 and T2−T0 represent the relative differences between the 2-week and 1-month assessment results, respectively, compared to the baseline.

**9. Comparison of model parameters between active and sham groups: analysis of variance results**


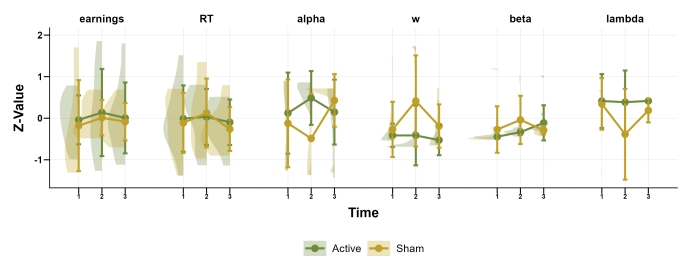


Figure S7 Estimated marginal means for the active group and sham group across time points.  Error bars indicate standard error. The y-axis depicts the standardised values of behavioural measures and model parameters, while the x-axis represents the times of measurements. The colors discriminate between the active-treatment group and the sham group.

alpha numDF denDF F-value p-value

(Intercept) 1 40 0.000000 1.0000

factor(time) 2 40 0.000000 1.0000

invgroup 1 20 0.724447 0.4048

factor(time):invgroup 2 40 4.305162 0.0203

Value Std.Error DF t-value p-value

(Intercept) 0.1223093 0.2923650 40 0.4183446 0.6779

factor(time)2 0.3645474 0.3395620 40 1.0735811 0.2894

factor(time)3 -0.3398618 0.3395620 40 -1.0008828 0.3229

invgroupSham -0.2446186 0.4134665 20 -0.5916286 0.5607

factor(time)2:invgroupSham -0.7290948 0.4802133 40 -1.5182729 0.1368

factor(time)3:invgroupSham 0.6797237 0.4802133 40 1.4154621 0.1647

beta numDF denDF F-value p-value

(Intercept) 1 40 0.000000 1.000

factor(time) 2 40 0.000000 1.000

invgroup 1 20 0.271570 0.608

factor(time):invgroup 2 40 3.585249 0.037

Fixed effects: value ~ factor(time) * invgroup

Value Std.Error DF t-value p-value

(Intercept) -0.1951778 0.2961557 40 -0.6590377 0.5136

factor(time)2 -0.1392954 0.3445246 40 -0.4043121 0.6881

factor(time)3 0.4822733 0.3445246 40 1.3998227 0.1693

invgroupSham 0.3903555 0.4188274 20 0.9320200 0.3624

factor(time)2:invgroupSham 0.2785909 0.4872313 40 0.5717836 0.5707

factor(time)3:invgroupSham -0.9645466 0.4872313 40 -1.9796483 0.0547

**ω**  numDF denDF F-value p-value

(Intercept) 1 40 0.0000000 1.0000

factor(time) 2 40 0.0000000 1.0000

invgroup 1 20 2.6293127 0.1206

factor(time):invgroup 2 40 0.8827513 0.4215

Value Std.Error DF t-value p-value

(Intercept) -0.1768784 0.2977221 40 -0.5941057 0.5558

factor(time)2 -0.2377546 0.4076269 40 -0.5832654 0.5630

factor(time)3 0.1411473 0.4076269 40 0.3462659 0.7310

invgroupSham 0.3537568 0.4210426 20 0.8401924 0.4107

factor(time)2:invgroupSham 0.4755093 0.5764714 40 0.8248618 0.4143

factor(time)3:invgroupSham -0.2822945 0.5764714 40 -0.4896939 0.6270

**λ**  numDF denDF F-value p-value

(Intercept) 1 40 0.0000000 1.0000

factor(time) 2 40 0.0000000 1.0000

invgroup 1 20 2.4954035 0.1299

factor(time):invgroup 2 40 0.6095705 0.5486

Value Std.Error DF t-value p-value

(Intercept) 0.2188968 0.2975696 40 0.7356154 0.4663

factor(time)2 0.1665369 0.3629900 40 0.4587920 0.6489

factor(time)3 -0.1153207 0.3629900 40 -0.3176966 0.7524

invgroupSham -0.4377936 0.4208270 20 -1.0403173 0.3106

factor(time)2:invgroupSham -0.3330739 0.5133454 40 -0.6488299 0.5202

factor(time)3:invgroupSham 0.2306413 0.5133454 40 0.4492908 0.6556

1. **Longitudinal changes in central theta oscillatory dynamics under different feedback conditions.**

**10.1 Plotting**

**
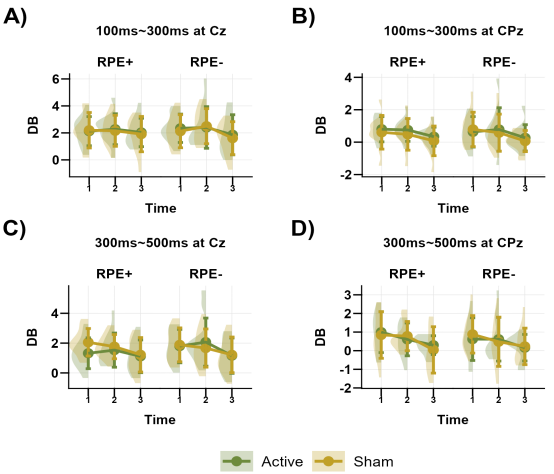
**

Figure S8 Longitudinal changes in central theta oscillatory dynamics under different feedback conditions. The y-axis depicts the values of theta power in unit of DB, while the x-axis represents the measurement times. The error bar indicates standard error. The colors discriminate between the active-stimulation group and the sham group. The alterations in power dynamics between 100ms and 300ms are highlighted in A) and B) for the Cz and CPz channels, respectively. Similarly, C) and D) showcase the power fluctuations between 300 ms and 500 ms at the Cz and CPz channels.

**10.2 Comparison of theta power at 100–300ms and 300–500ms between active and sham groups: analysis of variance results**

100ms~300ms at Cz numDF denDF F-value p-value

(Intercept) 1 60 219.20247 <.0001

factor(wave) 2 60 0.98768 0.3784

invgroup 1 60 0.09895 0.7542

condition 1 60 0.04632 0.8303

factor(wave):invgroup 2 60 0.03170 0.9688

factor(wave):condition 2 60 1.60119 0.2102

invgroup:condition 1 60 0.09444 0.7597

factor(wave):invgroup:condition 2 60 0.45121 0.6390

300ms~500ms at Cz numDF denDF F-value p-value

(Intercept) 1 60 146.50003 <.0001

factor(wave) 2 60 2.27317 0.1118

invgroup 1 60 0.27623 0.6011

condition 1 60 1.23312 0.2712

factor(wave):invgroup 2 60 0.31460 0.7313

factor(wave):condition 2 60 0.24733 0.7817

invgroup:condition 1 60 3.44986 0.0682

factor(wave):invgroup:condition 2 60 0.68550 0.5077

100ms~300ms at Cz numDF denDF F-value p-value

(Intercept) 1 60 23.215890 <.0001

factor(wave) 2 60 2.379916 0.1012

invgroup 1 60 0.570370 0.4531

condition 1 60 0.015365 0.9018

factor(wave):invgroup 2 60 0.112643 0.8937

factor(wave):condition 2 60 0.130205 0.8782

invgroup:condition 1 60 0.824430 0.3675

factor(wave):invgroup:condition 2 60 0.284801 0.7532

300ms~500ms at Cpz numDF denDF F-value p-value

(Intercept) 1 60 219.20247 <.0001

factor(wave) 2 60 0.98768 0.3784

invgroup 1 60 0.09895 0.7542

condition 1 60 0.04632 0.8303

factor(wave):invgroup 2 60 0.03170 0.9688

factor(wave):condition 2 60 1.60119 0.2102

invgroup:condition 1 60 0.09444 0.7597

factor(wave):invgroup:condition 2 60 0.45121 0.6390

**10.3 TF plot for active and sham group changed theta powers at Cz**


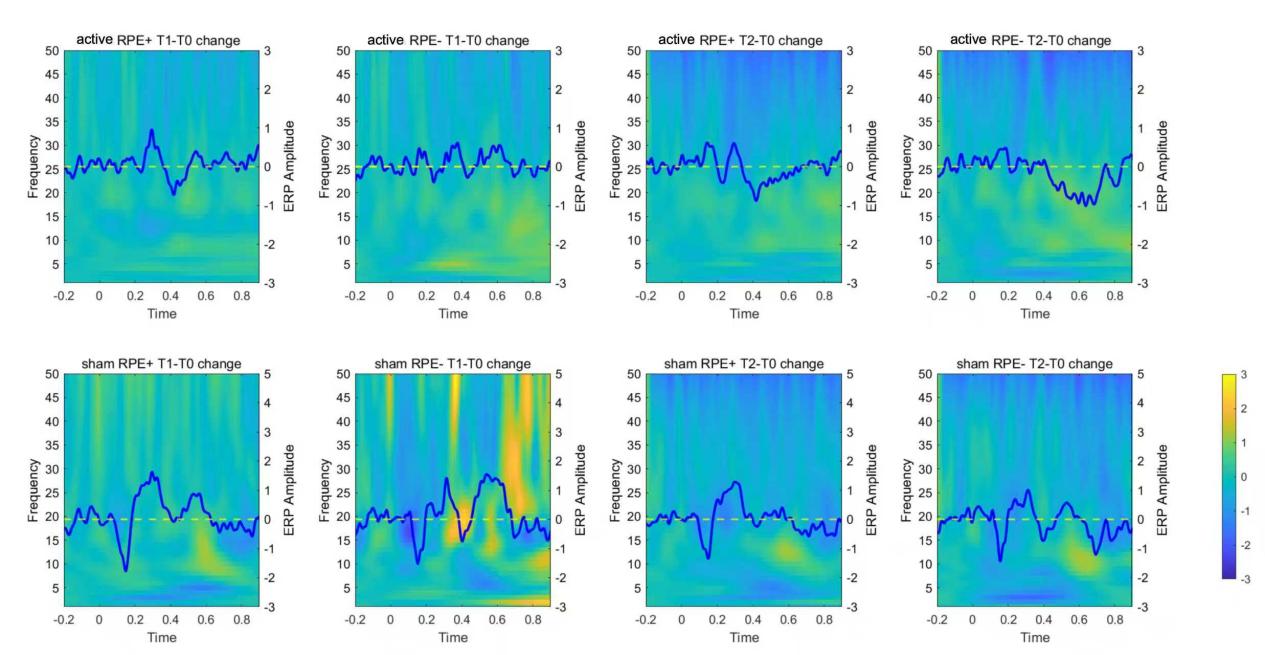


Figure S9 Event-related potentials and time-frequency characteristics for changes in theta powers at Cz for both the active and sham groups after positive and negative reward reward prediction errors.
